# Principal Component Analysis of Stair Negotiation and Floor Transition Kinematics in Older Adults With and Without Functional Disability: Cross-Sectional Study

**DOI:** 10.2196/71530

**Published:** 2025-08-27

**Authors:** Juliana Moreira, Ivone da Silva Teles, Bruno Cunha, José Félix, Diana C Guedes, Leonel A T Alves, Rubim Santos, Andreia S P Sousa

**Affiliations:** 1 CIR, E2S, Polytechnic of Porto, Rua Dr. António Bernardino de Almeida nº 400, Porto, 4200-072, Portugal, 351 222 061 000; 2 Research Center in Physical Activity, Health and Leisure, Faculty of Sports, University of Porto, Porto, Portugal; 3 CINTESIS@RISE, CINTESIS.UPT, Portucalense University, Porto, Portugal; 4 Department of Physiotherapy, Institute of Health of the North - Escola Superior de Saúde do Vale do Ave (ESSVA), Cooperativa de Ensino Superior Politécnico e Universitário (CESPU), Vila Nova de Famalicão, Portugal; 5 Department of Medical Sciences, University of Aveiro, Aveiro, Portugal

**Keywords:** disability, biomechanics, stair negotiation, gerontology, kinematics, factor analysis

## Abstract

**Background:**

Stair negotiation (ascending and descending) and transitions to level walking are complex motor tasks influenced by aging; yet the impact of functional disability on these changes remains underexplored.

**Objective:**

This study aimed to evaluate the lower limb joint positions, velocities, and the center of mass (CoM) displacement and velocity during stair negotiation and transitions in older adults with and without functional disability.

**Methods:**

Sixty community-dwelling adults, aged 60 years and older, were assessed for lower limb tridimensional joint positions and velocities during the instances of foot contact or leaving the step and foot contact or leaving the floor; the joint range of motion, angular velocity range, and the CoM displacement and angular velocity range were assessed during stair ascend and descend and transitions floor-to-stair and stair-to-floor through an optoelectronic system. Principal component analysis was used to assess 8 groups of variables to compute principal component models (I-VIII). Participants were classified as with or without disability based on functional disability indicators. Group differences were assessed using the Mann-Whitney *U* test.

**Results:**

From 240 variables, 41 key parameters were identified, mainly related to hip and knee angular velocities in the sagittal plane. Significant differences between older adults with disability (n=25) and without disability (n=35) were found in 12 principal components.

**Conclusions:**

Older adults with functional disability showed changes in the sagittal plane hip as well as in the knee angular velocity and mediolateral and vertical CoM displacement and velocity during stair negotiation and transitions. These findings can inform targeted strategies to improve mobility and stability in this population.

## Introduction

Disability, as described by the International Classification of Functioning, Disability, and Health, embraces the impairments, activity limitations, and participation restrictions [1]. It is a condition that requires assistance to overcome reliance or difficulty with everyday tasks and independent living [2]. Aging is associated with systemic changes in the body, including deficits in cardiorespiratory function, slowness, strength loss, reduced flexibility, and weight loss, resulting in a decreased ability to perform daily activities [3,4]. These alterations increase the risk of mobility limitations [4] and physical disability [2] in older adults, making it crucial to reduce the length of handicap time and increase the well-being and independent time [5]. Research and development investment is fundamental for effective intervention strategies, especially in the rapidly aging global population, requiring policy makers to anticipate these changes [5]. Accordingly, to delay disability or prevent its occurrence, a multidimensional assessment needs to be performed [6]. One hallmark of disability and functional decline in older adults is related to impairments in stairs negotiation, the act of ascending or descending stairs [7]. These tasks are associated with a high risk of mortality and serious injuries [8], with estimates that 20% of falls occur on stairs [9], making them 10 times more dangerous than walking [10]. The fact that this is one of the most hazardous daily activities is justified by its motor complexity, which, requiring precise coordination of joint movements, muscle forces, and balance control, poses a significant challenge for older adults [11], particularly those with functional disability [12].

The association between aging and biomechanical performance during stair negotiation has been reported in previous studies [9,10,13-15]. Specifically, older adults seem to present slower cadence and higher variability of hip joint angle curves in the sagittal plane during stair ascent and descent [13,16]. They also present higher maximum dorsiflexion angles [17] and increased time to descend stairs, which has been considered a predictor of functional decline [18]. A reduction of peak downwards and upwards center of mass (CoM) acceleration during the descent and landing phases, respectively [19], was also identified in older adults’ performance.

While age-related biomechanical adaptations in stair negotiation are well-documented, the extent to which these are associated with disability, as opposed to healthy aging, remains underexplored. Moreover, stair negotiation assessment should also encompass the sequences of walking to ascend stairs and descending stairs to walk, to represent the complete range of this daily activity. Therefore, it is important to evaluate not only stair use (stair ascent and descent) but also floor transitions (floor-to-stair [F-S] and stair-to-floor [S-F]). During stair use and transitions, beyond the range of kinematic parameters during full cycles of stair ascent and descent as well as F-S and S-F, the assessment of key events could bring relevant data because at these transitional moments, older adults demonstrated more impairments in margin of stability [20], lower downwards CoM velocity [19], and lower peak moments during stair ascent and descent [17]. These changes lead to compensatory strategies prioritizing stability over range of motion (ROM), particularly when making or leaving contact with the step [17]. Additionally, anatomical changes related to aging such as sagittal malalignment, were associated with ascending and descending stairs with increased thorax flexion, increasing the fall risk [21], and previous research highlighted the roles of pelvis and head kinematics [22].

Addressing this research gap can provide valuable insights into the biomechanical contributors to stair negotiation impairments. Moreover, the lack of research is even more pronounced in studies comparing older adults with functional disability to those experiencing healthy aging. Such investigations can inform the development of targeted interventions to improve stair performance, reduce fall risk, and enhance functional independence in older populations.

Principal component analysis (PCA), a multivariate and nonparametric statistical technique, reduces the dimensionality of biomechanical data by generating a new set of uncorrelated, orthogonal variables known as principal components (PCs) [23,24]. It has been used to analyze biomechanical data from older adults during stair negotiation, primarily in comparisons with younger adults [14].

Following this rationale, this study examines the kinematic differences in lower limb joint position and velocity at the instant of contact and leaving the step, the joint ROM, the variation of angular velocity range (Δvelocity), and CoM displacement and velocity variation during stair ascent and descent cycles in older adults with and without disabilities. Additionally, it investigates the same kinematic variables at contact and leaving the floor moments, and F-S and S-F cycles.

## Methods

### Study Design

A cross-sectional study following the recommendations of the STROBE (Strengthening the Reporting of Observational Studies in Epidemiology) [25] guideline was designed and implemented as part of an observational registered study in the ClinicalTrials.gov database (NCT05611723). Between June 1, 2022, and March 31, 2023, a sample selection questionnaire was distributed to the partners of Center for Rehabilitation Research of the School of Health Polytechnic of Porto, where this study took place. The data collection continued until April 30, 2023.

### Participants

Individuals were enrolled in this study if they were aged older than 60 years, community residents of the northern region of Portugal, and independently capable of completing the tasks (walking, sitting, and standing from a chair, and ascending and descending stairs). The following conditions precluded volunteers from participating in this study: institutionalization; had an established diagnosis of malignancy or terminal diseases with an anticipated survival of less than 1 year; had stroke history, cerebral hemorrhage, head trauma, or Parkinson disease; had cognitive impairment, specifically Mini-Mental State Examination (MMSE, Portuguese version) score of 22 for 0 to 2 years of literacy, 24 for 3 to 6 years of literacy, and 27 for more than 6 years of literacy [26]; had rheumatic pathologies that interfere with the tasks performance; had diabetic foot, lower limb fracture in the past 6 weeks, or other related conditions; had unstable cardiovascular disease, liver or renal function failure; had a BMI greater than 30 kg/m^2^; had uncorrected vestibular or audition or vision impairments that could influence the performance of tasks; or had symptoms at the time of evaluation that could influence task performance.

### Procedures and Instruments

#### Sample Selection and Characterization

A questionnaire was used to characterize the participants regarding demographic data, health conditions, and history of falls in the past 12 months. Additionally, participants were characterized regarding anthropometric parameters (height, weight, and BMI), cognitive function (MMSE), self-reported medication intake, and self-reported physical activity (International Physical Activity Questionnaire: Short Form—Portuguese version).

Anthropometric parameters, specifically body mass (kg), were obtained by bioimpedance assessment (Tanita Inner Scan BC-601), with a precision of 0.1 kg. Height was measured using the seca 222 stadiometer (seca—Medical Scales and Measuring Systems), with a 1 mm scale. The MMSE (Folstein et al, 1975 [27]) was applied to assess the cognitive function of the participants. The total score ranges from 0 to 30, with a score of 22 for literacy from 0 to 2 years, 24 for literacy from 3 to 6 years, and 27 for literacy of 7 years or more [26]. Medication intake was self-reported, and polypharmacy was defined as taking 5 or more prescription drugs [28]. Self-reported physical activity was assessed by the 7-item short version of International Physical Activity Questionnaire [29].

#### Disability Indicators Characterization and Assessment

Participants were divided into 2 groups: older adults without disabilities and those with 2 or more disability indicators of the previously identified in the literature [30] namely: the handgrip strength (hand-held dynamometer), balance (single leg balance test time), overall self-reported health status (SRH; self-reported question), basic activities of daily living (ADL; Barthel Index) and instrumental activities of daily living (IADL; Lawton and Brody).

The handgrip strength was measured using a handheld dynamometer Jamar Plus+ Digital (Performance Health Supply). For the assessment, participants were seated with the shoulder adducted, the elbow flexed at 90 degrees and unsupported, the forearm neutral, and the wrist at 30 degrees of extension, as advised by the American Society of Hand Therapists [31]. The total time spent standing was measured in seconds using the participants’ favored leg and with eyes open. The test was completed when the participants could not maintain the position or reached the maximum duration of 60 seconds. This time was used to determine when the individual became imbalanced (eg, flexed leg contacting the ground) [32].

By responding to the query “In general, how do you rate your health today?,” the overall SRH was evaluated as (“very bad,” “bad,” “fair,” “good,” and “very good.” Good SRH (defined as “very good” or “good”) and poor SRH (defined as “fair,” “bad,” or “very bad”) were dichotomized [33]. The participant’s level of independence in the basic ADL was assessed using the Barthel Index [34]. The scoring system, ranging from 0 (complete dependency) to 20 (functional independence), was used as this scoring corrects for an uneven impression of accuracy [35]. The Lawton and Brody [36] IADL Scale was used to assess the functional capacity of older adults. It assesses 7 instrumental activities, and the final score ranges from 7 to 23, corresponding to the maximum score of total independence [37].

#### Kinematic Assessment

The stair negotiation was recorded using the Qualisys Track Manager (Qualisys AB), with 12 optoelectronic cameras (8 Oquos500 and 3 MiqusM3), 1 Miqus video camera, and 2 force plates (FP4060-08/10, Bertec). Kinematic data were collected at a 100 Hz frequency.

**Figure 1.**
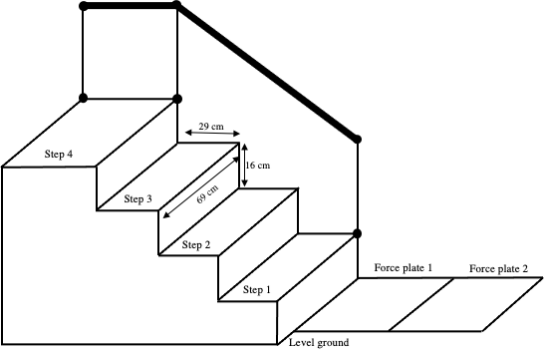
Scheme of stairs ascent, descent, and transitions with the floor setup.

Stair negotiation was performed in a 4-step staircase (step height: 16 cm, depth: 29 cm, and width: 69 cm; Figure 1). The topmost step of the staircase was labeled “step 4,” and the subsequent steps were labeled “step 3,” “step 2,” and “step 1.” To successfully complete the stair ascent, participants had to stand in front of the staircase before the force plates, take the first step on level ground on the force plate 2, the second step on the force plate 1, and then ascend 3 stairs using a step-over-step sequence at their own leisure [13]. Each participant was informed on how to descend the staircase, which included starting at step 4 in an upright stance with their feet side by side and moving down on their own self-selected initiating limb and velocity [19,38]. Additionally, participants were told to keep walking over the force plates for at least 2 steps after they reached level ground [39]. When participants reached a standing position on level ground, the trial was considered over [19]. Throughout the testing, handrails were installed as a safety measure; however, participants were told not to use them unless absolutely essential. A total of 3 valid trials were recorded for each participant [10].

### Data Processing

#### Overview

Qualisys Track Manager (Qualisys AB; version 2020.3) was used to process marker trajectories of the 3 trials per participant. The data was then exported to Visual3D Professional (Has-Motion, Inc) [40].

The joint angles were calculated using the Cardan sequence that assumes that the x-axis is in the medial-lateral direction, the y-axis is anterior-posterior, and the z-axis is in the up and down or axial direction.

To characterize the sequences of walking to ascending and descending stairs to walk, the values from cycle time (s), speed (m/s), and peak vertical velocity from the CoM (absolute value in meters per second) were computed.

**Figure 2.**
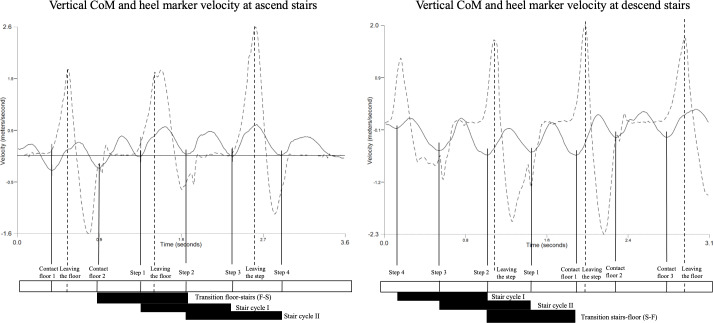
Events definition for each task, stair ascent and stair descent, and respective transitions with the floor F-S and S-F. The regular line represents vertical CoM velocity, and the dashed line represents the vertical heel marker velocity. CoM: center of mass; F-S: floor-to-stair transition; S-F: stair-to-floor transition.

The complete daily activity sequences of walking to ascend stairs and descending stairs to walk were divided into subtasks, specifically stair use (stair ascent and descent) and floor transitions (F-S and S-F). Thus, 2 distinct cycles were identified for stair ascent and descent. For the ascending stairs task, 1 stair cycle began in step 1 and ended in step 3, and another began in step 2 and ended in step 4. The descending task cycles were defined in the opposite direction (Figure 2). The F-S was considered as the instant the foot left the floor and ended when the foot left the step, with the opposite transition (S-F) following the reverse pattern (Figure 2). The moment of leaving the step or the ground was defined by the maximum heel marker’s vertical velocity, and the moment of contact with the step or the ground was determined by the minimum downward CoM velocity [41].

#### Processing Joint Position and Velocities at Events

During both stair cycles and the S-F and F-S, data on position and angular velocity of the hip, knee, and ankle joints were extracted from the ipsilateral limb performing the contact with the step or floor and the moment the foot left the step or floor. Accordingly, the variables selected were position and angular velocity of hip, knee, and ankle according to the 3 axes (X, Y, Z) at the contact with the step and leaving the step at ascent and descent stairs, and the contact with the floor and leaving the floor at the F-S and S-F.

#### Processing Joint Ranges of Motion and Velocities, CoM Displacement and Velocity Range at Cycles

The ROM was calculated as the difference between the maximum and minimum lower limb angles during the stair ascent and descent cycles and F-S and S-F cycles. The difference between the maximum and minimum angular velocities of the lower limb joints was defined as Δvelocity. The COM was computed by incorporating all body segments, with segment lengths determined by joint centers, and its position had as a resolution coordinate system the laboratory coordinate system, and the CoM velocity by taking the derivative of the CoM position over time [42]. The CoM displacement and CoM Δvelocity in the 3 planes (X, Y, Z) were also computed for both the stair cycles and the S-F and F-S. All the computations were performed in the software Visual3D Professional.

### Statistical Analysis

The statistical software G*Power (version 3.1.9.7; Heinrich-Heine-Universität Düsseldorf) was used to determine a 0.948 power (1-β), introducing as independent variables the significance level (*α*=.05), effect size (*f*=0.6667), and sample size (n=60) [43].

The IBM SPSS (version 28.0; SPSS Inc) statistical package was used to compile the data and for statistical analysis. The normality of the distribution was assessed by the Kolmogorov-Smirnov and Shapiro-Wilk tests for the group of older adults without and with disability, respectively. Between-group comparisons of demographic and clinical characteristics were conducted using Mann-Whitney tests or an independent sample *t* test (2-tailed). *χ*^2^ tests were used to evaluate between-group association in the distribution of the categorical variables, namely sex, history of fall, and SRH.

**Figure 3.**
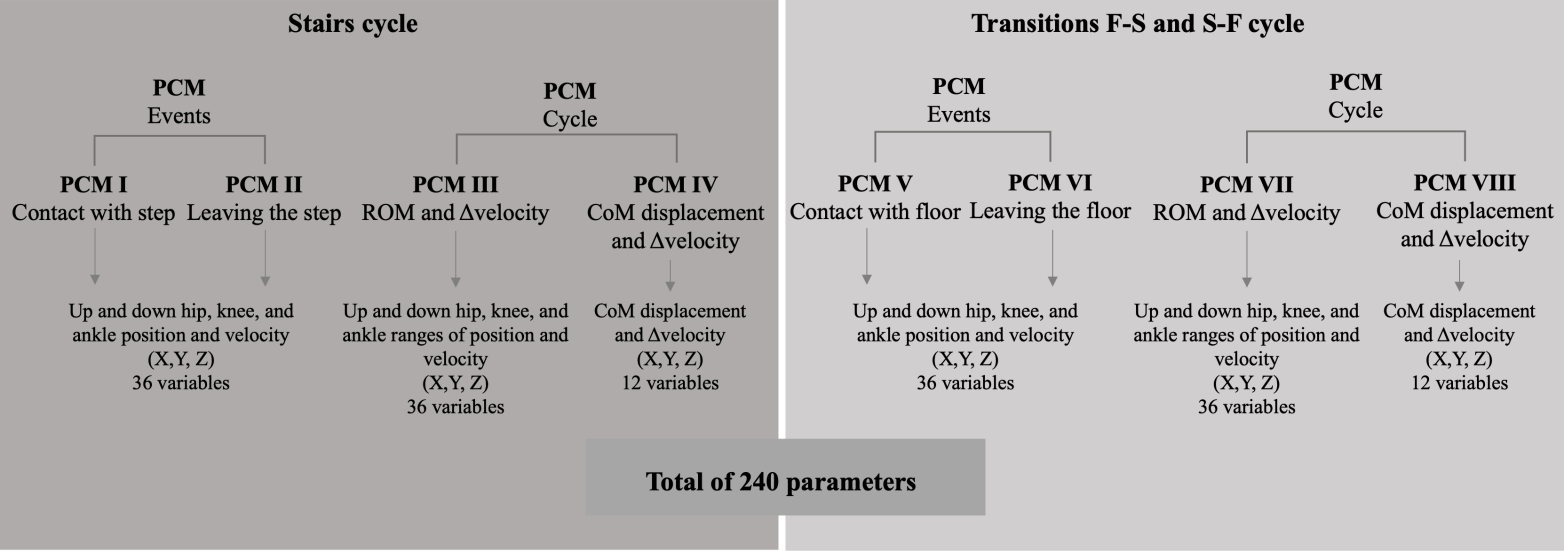
Parameters included in each PCM, divided by the subtasks, stairs, and transitions with the floor. CoM: center of mass; F-S: floor-to-stair transition; PCM: principal component model; ROM: range of motion; S-F: stair-to-floor transition; Δvelocity: angular velocity range.

Individual principal component models (PCMs) were made for different sets of variables. Particularly, PCM I included the position and velocity of the hip, knee, and ankle on the 3 planes (X, Y, Z) at the moment of contact with the step at stairs ascending and descending, and PCM II the same parameters at the moment the foot left the step during stair ascending and descending. The PCM V and VI included the same set of variables at contact with floor and leaving the floor moments, respectively, during the S-F and F-S. PCM III was computed for ROM and Δvelocity of hip, knee, and ankle on the 3 planes at the stairs ascent and descent, and PCM VII for the same set of variables at S-F and F-S. Additionally, PCMs IV and VII were conducted for COM displacement and Δvelocity on stairs and transition to floor cycles, respectively (Figure 3). PCA was carried out utilizing the correlation matrix to guarantee that every variable contributed equally to the analysis; accordingly, *z* scores were used to account for the varying measurement scales of the variables. The correlation matrix is based on standardized values, meaning that each variable was divided by its SD, effectively normalizing the variables to have a mean of 0 and an SD of 1. This step ensures that variables with different units and every variable contribute equally to the analysis. The suitability of the dataset for PCA was evaluated using Kaiser-Meyer-Olkin (KMO), as a measure of sampling adequacy for the overall data, and Bartlett Test of Sphericity as a measure of data suitability for reduction. A significant Bartlett test (*P*<.05) and KMO values larger than 0.5 were used to determine whether the data were suitable for PCA. Potential outliers were manually inspected in the data and verified not to unduly influence the PCA results. Using eigenvalues greater than 1 as the criterion, the PCs that accounted for most of the variance were extracted, ensuring that only meaningful variance contributors were retained for biomechanical interpretation. To aid in the interpretability of the components, a Varimax rotation was applied to redistribute variance across PCs, making it easier to identify biomechanical patterns. The rotated component matrix was examined to identify clusters of variables that loaded onto the same components. The components were interpreted based on the variables that exhibited high loadings (loading >0.7, as the sample is n=60 [44]) on each component. The PC scores were tested for normality using the Kolmogorov-Smirnov and Shapiro-Wilk tests. As the data did not follow a normal distribution, the nonparametric Mann-Whitney *U* test was applied to assess differences between older adults with and without disability indicators, with the significance level set at 0.05.

### Ethical Considerations

This study adhered rigorously to the principles outlined in the Declaration of Helsinki. All participants received information about this study's purpose and methodology, and they completed and signed an informed consent form. This study was submitted to the Institutional Ethics Committee of E2S, Polytechnic of Porto on April 27, 2022, and obtained authorization on May 25, 2022 (CE0064C). Participants’ identities and confidentiality were maintained throughout the research study. All participant data were deidentified and were stored on password-protected secure servers to prevent unauthorized access. There was no known risk or harm to participating in this study or publicizing its results or findings; participation was voluntary, and the participants did not receive compensation in this study.

## Results

### Sample and Tasks Characterization

**Figure 4.**
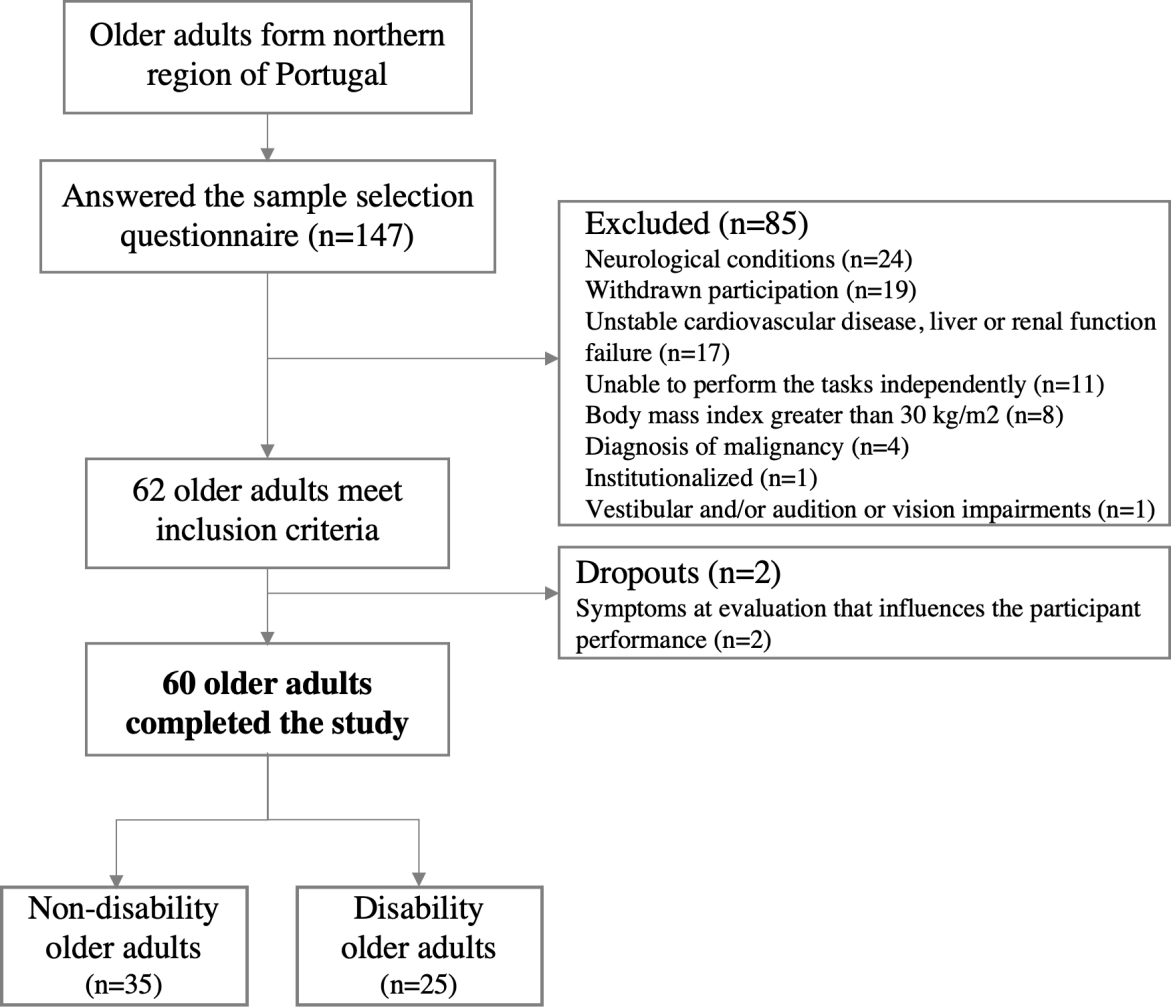
Flowchart of the sample selection process.

A total of 62 individuals, from 147 older adults, enrolled in the study, as shown in Figure 4. During the evaluation, 2 participants exhibited symptoms that affected the activities performance. As a result, 60 participants were included in the analysis of this study. Table 1 provides a summary of the descriptive statistics of their clinical and demographic features.

In examining the factors serving as markers for disability, substantial differences were observed between groups with and without disability (Table 1). Older individuals with disabilities exhibited poorer independence in ADL and IADL (*P*=.01 and *P*=.01, respectively), as well as reduced hand grip strength (*P*=.018) and diminished time spent in a 1-leg stand posture (*P*<.001). Furthermore, in comparison to the nondisabled group, older persons with disabilities rated their health as poorer (*P*<.001). Polypharmacy also showed significant disparity between the groups (*P*<.001).

Regarding stair negotiation, older adults with disabilities demonstrated a longer cycle time (*P*=.003), reduced speed (*P*<.001), and lower peak vertical velocity of the CoM (*P*=.003) while ascending stairs (Table 2). In the descending stairs task, older adults with disabilities exhibited lower speed (*P*<.009) and CoM peak velocity (*P*<.013).

**Table 1.** Demographic and clinical characterization of the sample. The *P* value reflects the comparison between ND^a^ and D^b^.

	ND (n=35)	D (n=25)	*P* value	Test value
Age (years)	66.34 (5.60)^c^	68.60 (6.77)^c^	.15	534^d^
Gender (female)	19 (54.29)^e^	19 (76)^e^	.09	2.9 (*df*=1)^f^
BMI (kg/m^2^)	25.22 (3.08)^c^	26.02 (2.66)^c^	.30	−1.049^g^
History of falls in the previous 12 months (fallers)	11 (31.4)^e^	11 (44)^e^	.47	0.5 (*df*=1)^f^
Polypharmacy (polymedicated)	2 (5.71)^e^	11 (44)^e^	<.001	12.5 (*df*=1)^f^
Cognitive function (MMSE^h^ score)	28.94 (1.31)^c^	28.68 (1.49)^c^	.50	394^d^
Self-reported physical activity (IPAQ^i^ MET^j^-min/wk)	3186.46 (2964.91)^c^	3519.66 (2822.11)^c^	.51	393.5^d^
Self-reported health			<.001	21.8 (*df*=1)^f^
Poor	8 (22.86)^e^	21 (84)^e^		
Good	27 (77.14)^e^	4 (16)^e^		
Hand grip strength (kg)	36.59 (39.86)^c^	25.07 (7.54)^c^	.02	279.5^d^
One leg standing time (s)	38.83 (20.93)^c^	18.19 (20.72)^c^	<.001	192.5^d^
ADL^k^ independence (Barthel Index score)	19.97 (0.17)^c^	19.76 (0.44)^c^	.01	345^d^
IADL^l^ independence (Lawton & Brody score)	23 (0.00)^c^	21.96 (2.67)^c^	.01	332.5^d^

a ND: older adults without disability.

b D: older adults with disability.

c Mean (SD).

d Mann-Whitney *U* test

e n (%).

f *χ*2 test

g Independent samples *t* test (2-tailed).

h MMSE: Mini-Mental State Examination.

i IPAQ: International Physical Activity Questionnaire.

j MET: metabolic equivalent of task.

k ADL: activities of daily living

l IADL: instrumental activities of daily living.

**Table 2.** Walk to ascending stairs and descend stairs to characterize walk tasks according to cycle time, double limb support time, speed, and CoM^a^ peak velocity mean and SD. The *P* value reflects the comparison between ND^b^ and D^c^.

	Walking to ascend stairs				Descending stairs to walk			
	ND, mean (SD)	D, mean (SD)	*P* value	Test value	ND, mean (SD)	D, mean (SD)	*P* value (test value)	Test value
Cycle time (s)	1.15 (0.23)	1.31 (0.16)	.003	−2.876^d^	1.13 (0.21)	1.22 (0.16)	.06	562.0^e^
Speed (m/s)	0.68 (0.11)	0.60 (0.08)	<.001	3.278^d^	0.86 (0.18)	0.76 (0.14)	.009	2.450^d^
CoM peak vertical velocity (m/s)	0.20 (0.05)	0.17 (0.03)	.003	−2.853^d^	0.56 (0.07)	0.52 (0.08)	.013	−2.284^d^

a CoM: center of mass.

b ND: older adults without disability.

c D: older adults with disability.

d Independent samples *t* test (2-tailed).

e Mann-Whitney *U* test

### PCA Models

PCMs I to VIII presented KMO values above 0.5, and Bartlett Test of Sphericity was significant (*P*<.001) for all PCMs, indicating that the data were suitable for dimensionality reduction. Table S1 in Multimedia Appendix 1 includes a detailed description of the PCs with eigenvalues greater than 1 for each of the PCMs.

This study observed statistically significant differences (*P*<.05) between older adults with and without disabilities in 12 PCs across PCM I to VIII. After applying Varimax rotation, the components were interpreted based on the variables with loadings exceeding 0.7, as outlined in Table 3. Analysis of the variables with higher loadings in the PCs revealed a prevalence of variables associated with angular velocity, distributed similarly during ascending and descending tasks, particularly in the sagittal plane. Notably, the contact of the foot with the step was found to be important in the ascending task regarding frontal hip angular velocity and sagittal knee velocity (PCM I).

**Table 3.** Variables with loadings higher than 0.7 of the PC^a^ that presented significant differences between ND^b^ and D^c^, with respective mean and SD of each group. The tasks ascending stairs and descending stairs are designated up and down, respectively.

PCM^d^	PC	Variable	Load	ND, mean (SD)	D, mean (SD)
Stairs					
PCM I (contact with step)	4				
		Up hip velocity (Y)	0.814	18.7 (22.0)	10.3 (20.5)
		Up knee velocity (X)	0.888	−140.2 (62.2)	−89.1 (42.9)
PCM II (leaving the step)					
	2	Up hip angle (X)	0.750	34.7 (12.7)	30.4 (11.4)
	2	Up hip velocity (X)	0.784	199.7 (43.2)	168.7 (36.1)
	2	Up knee angle (X)	0.811	58.4 (11.6)	49.7 (12.7)
	2	Up knee velocity (X)	0.847	392.5 (91.4)	336.5 (63.9)
	2	Up ankle velocity (X)	0.744	128.0 (87.0)	84.9 (79.4)
	3	Down hip velocity (X)	0.804	153.0 (43.4)	113.5 (35.1)
	3	Down knee velocity (X)	0.877	283.2 (86.2)	197.0 (87.3)
	3	Down ankle velocity (X)	−0.853	−212.6 (50.8)	−160.0 (58.8)
PCM III (up and down cycles)					
	1	Up hip Δvelocity^e^ (X)	0.856	316.5 (45.4)	286.3 (35.1)
	1	Up knee Δvelocity (X)	0.901	651.1 (112.3)	587.0 (83.8)
	1	Down hip ROM^f^ (X)	0.803	27.8 (4.0)	27.0 (3.0)
	1	Down hip Δvelocity (X)	0.853	295.8 (85.4)	239.5 (45.4)
	1	Down knee Δvelocity (X)	0.807	690.6 (138.0)	570.7 (97.2)
	10	Down ankle ROM (Y)	0.867	12.4 (2.8)	14.4 (3.3)
PCM IV (up and down cycles)					
	1	Up CoM^g^ displacement (X)	0.824	0.05 (0.01)	0.04 (0.01)
		Up CoM Δvelocity (X)	0.855	0.22 (0.05)	0.19 (0.04)
		Down CoM displacement (X)	0.820	0.05 (0.01)	0.04 (0.01)
		Down CoM Δvelocity (X)	0.801	0.31 (0.06)	0.30 (0.05)
F-S^h^ and S-F^i^					
PCM V (contact with floor)	3				
		S-F hip velocity (Y)	0.852	7.2 (21.3)	−8.7 (15.0)
		S-F hip velocity (Z)	−782	−12.3 (23.2)	−5.4 (18.8)
		S-F knee velocity (Z)	0.841	10.3 (40.0)	−8.8 (28.9)
PCM VI (leaving the floor)					
	2	F-S ankle velocity (Z)	−0.746	4.7 (10.3)	−4.8 (16.6)
	2	S-F hip velocity (Z)	0.788	−14.6 (35.1)	0.7 (29.5)
	2	S-F ankle velocity (X)	0.718	−230.4 (61.8)	−168.0 (63.1)
	2	S-F ankle velocity (Z)	−0.816	4.2 (32.7)	−10.9 (30.1)
	3	F-S hip velocity (X)	−0.857	142.2 (34.0)	130.0 (24.2)
	3	F-S knee velocity (X)	−0.929	234.3 (116.5)	192.1 (97.6)
	9	F-S hip angle (Y)	0.784	1.8 (2.9)	3.7 (2.4)
	9	S-F hip angle (Y)	0.901	3.1 (3.4)	4.8 (3.4)
PCM VII (F-S and S-F cycles)	1				
		S-F hip ROM (X)	0.747	46.1 (7.0)	46.3 (6.0)
		S-F hip Δvelocity (X)	0.861	296.6 (63.6)	274.7 (50.1)
		S-F knee ROM (X)	0.789	75.5 (4.4)	73.4 (4.4)
		S-F knee Δvelocity (X)	0.888	750.6 (130.3)	652.7 (114.1)
		F-S hip Δvelocity (X)	0.865	329.4 (59.1)	297.7 (47.4)
		F-S knee ROM (X)	0.795	70.2 (6.6)	67.7 (5.7)
		F-S knee Δvelocity (X)	0.907	658.5 (122.5)	575.0 (91.6)
PCM VIII (F-S and S-F cycles)	2				
		Up CoM displacement Z	0.710	0.22 (0.01)	0.22 (0.01)
		Up CoM Δvelocity Z	0.833	0.60 (0.10)	0.65 (0.08)
		Down CoM Δvelocity Z	0.798	0.31 (0.06)	0.30 (0.05)

a PC: principal component.

b ND: older adults without disability.

c D: older adults with disability.

d PCM: principal component model.

e Δvelocity: angular velocity range.

f ROM: range of motion.

g CoM: center of mass.

h F-S: floor-to-stair transition.

i S-F: stair-to-floor transition.

Additionally, significant differences between groups were demonstrated in 2 PCs at the moment of leaving the step (PCM II), 1 defining the ascending (PC2) and the other the descending (PC3) tasks. Position and angular velocity on the sagittal plane of the hip, and knee and ankle angular velocity are present in PC 2, with higher loadings for the ones related to the knee joint. The PC3, defining the descending task, retained the hip, knee, and ankle angular velocities in the sagittal plane. The ROM and the range of angular velocities were divided into the sagittal and frontal planes, with PC1 retaining sagittal hip ROM and Δvelocity, and knee Δvelocity when descending stairs and hip and knee Δvelocity when ascending stairs. The PC 10 retained the frontal plane ROM of the ankle joint during descending (PCM III).

**Figure 5.**
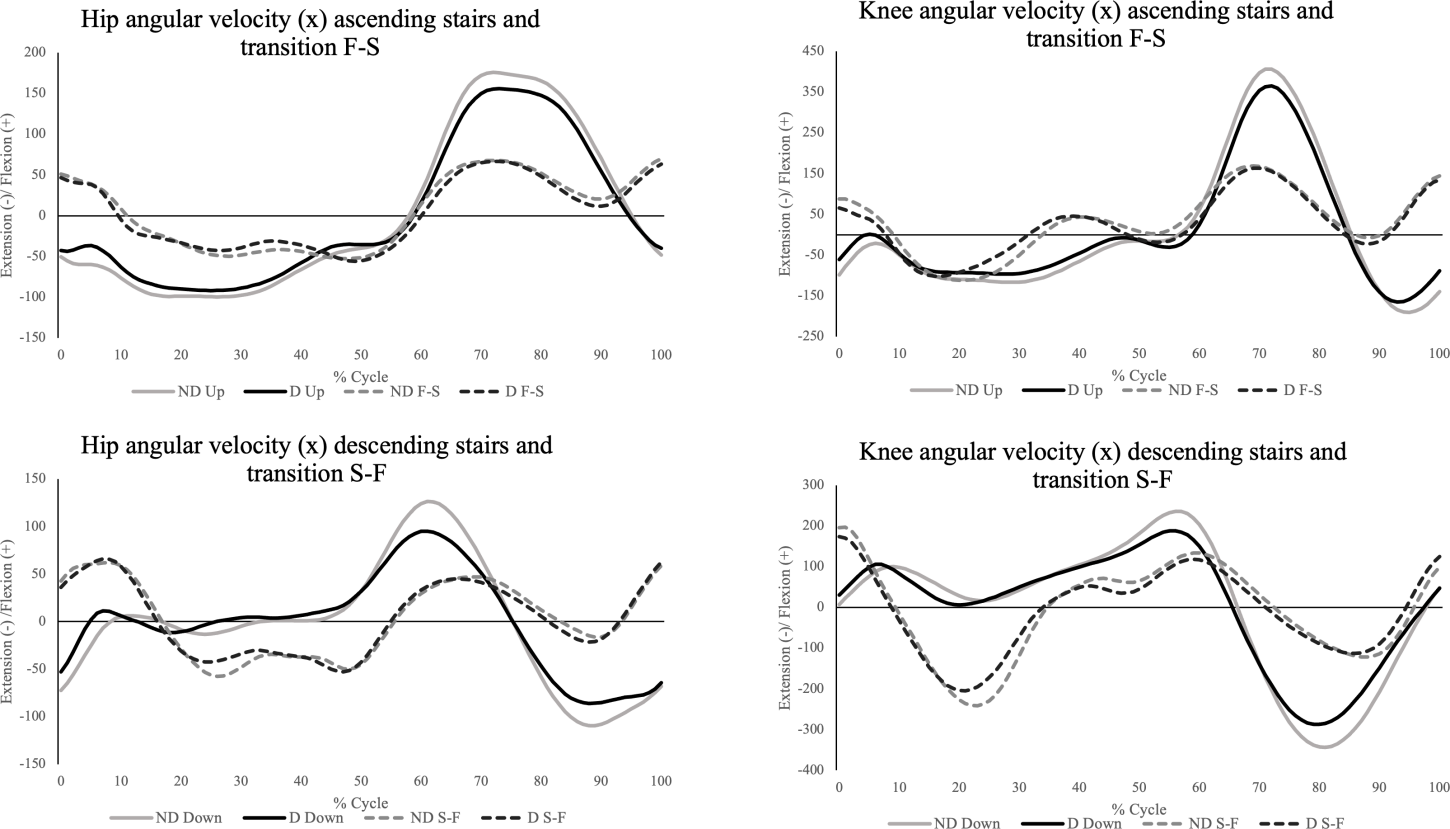
Sagittal hip and knee angular velocities on ascending stairs (up) and F-S and descending stairs (down) and S-F, within the cycle for ND and D. D: older adults with disability; F-S: floor-to-stair transition; ND: older adults without disability; S-F: stair-to-floor transition.

The PCM V regarding the contact moment retained only variables of the S-F, particularly, frontal and transverse hip angular velocity and transverse knee angular velocity. At the moment of leaving the floor (PCM VI), 3 PCs demonstrated differences between groups, gathering variables of S-F and F-S. PC2 presented higher loadings in angular velocities of the S-F of the transverse plane in the hip joint and sagittal and transverse planes in the ankle joint. This component also presented higher loading for the transverse ankle velocity in the F-S. PC3 retained F-S sagittal hip and knee velocities, as PC 9 retained frontal hip angles of both F-S and S-F. The PCM VII demonstrated significant differences between groups only in PC1, which explained 25% of the total variance. The variables with higher loadings were all of the sagittal planes (S-F hip and knee ROM and Δvelocity, and F-S hip Δvelocity and knee ROM and Δvelocity). The variables with higher loadings were all of the sagittal plane (S-F hip and knee ROM and Δvelocity, and F-S hip Δvelocity and knee ROM and Δvelocity). In analyzing both stair ascent and descent cycles as well as transitional movements, the parameters most consistently preserved were the angular velocities of the hip and knee in the sagittal plane. Accordingly, the complete cycle for the groups without and with disability is plotted in Figure 5. Therefore, this figure highlights the main differences between the groups identified by the PCA.

The CoM displacement and Δvelocity PCM IV built with stairs cycle data demonstrated differences between older adults with and without disability in PC 1 explained approximately 33% of the variance and retained the displacement and Δvelocity of CoM in the sagittal plane, both in ascending and descending stairs. The PC2 of PCM VIII, computed with the CoM data of transition to floor cycles, presented differences between the groups, retaining the variables considering the transverse plane, except for CoM displacement in the S-F.

## Discussion

### Principal Results and Comparison With Prior Work

This study investigated the kinematic differences in joint angles, joint velocities, and CoM displacement during stair ascent, descent, and F-S and S-F among older adults with and without disability. This study is believed to be the first to delve into the distinguishing parameters of kinematics between older adults with and without disability when negotiating stairs and transitions to the floor. As hypothesized, older adults with disabilities demonstrated significant differences in characterization parameters during stair ascent to walk and stair descent to walk, particularly in cycle time, speed, and CoM vertical peak velocity when compared to their nondisabled counterparts.

In this study, the time taken to walk to ascend stairs was significantly longer in older adults with disability. This contrasts with previous research, such as the study by Oh-Park et al (2011) [18], which considered stair descent time, but not ascent time, as a predictor of functional decline. This difference may be attributed to the fact that the participants in the study by Oh-Park et al (2011) [18] commenced the task in front of the stairs, whereas the current study evaluated stair negotiation and the transition to the floor, beginning and ending with walking. This approach is more challenging and reflective of real-life activities, as highlighted in the study by Vallabhajosula et al (2012) [45]. In addition to a longer time to complete the ascending task, older adults with disabilities also demonstrated reduced speed and lower CoM peak velocity while walking to ascent stairs and descending stairs to walk. This is consistent with a more conservative strategy that could reduce the risk for a fall that was identified in older adults compared to younger adults [19], which seems to be augmented in older adults with disability.

In addition to the characterization parameters of the tasks, this study encompassed a comprehensive analysis of a wide array of variables, including those related to the position and velocity of lower limb joints, CoM displacement and velocity in 3 axes, and the description of 2 tasks: ascending and descending. These tasks were further subdivided into subtasks, particularly using stairs and transitioning to the floor, and were characterized at 2 distinct moments: contact with the step or floor and leaving the step or floor, along with their respective ranges. Given the complexity and multidimensionality of the dataset, a PCA approach was deemed appropriate. PCA was used to elucidate the shared variance of the parameters associated with stair negotiation, thereby identifying a simplified representation that consolidates the most significant parameters. This approach simplifies the analysis of stair negotiation in older adults with disability. While the mechanics of successful stair negotiation for healthy older adults have been extensively documented [8], studies focusing on older adults with disability have predominantly centered on measuring the time taken to complete tasks [18]. The present study sought to assess the aforementioned set of parameters to gain a comprehensive understanding of the behavior of older adults with disability during stair negotiation. Moreover, it is fundamental to consider different axes, as previous studies have shown that factors in the sagittal plane are crucial [13,16,17], and limited studies have compared older adults to younger individuals in terms of the frontal and transverse planes [46,47]. Furthermore, the PCA analysis was instrumental in reducing and analyzing the data curves of knee angles, moments, and forces in a study by Reid et al (2010) [14]. That decision was informed by existing literature, which has highlighted the direct relationship between functional impairment during stair negotiation and the knee joints of older adults compared to their younger counterparts [14]. In line with this rationale, a conservative approach was adopted in the present study, entailing the analysis of discrete data collected at different events as an initial step in exploring the task of stair negotiation among older adults with disability. By focusing on discrete events rather than continuous data, our PCM approach provides a more granular analysis of stair negotiation mechanics.

Accordingly, the use of individual PCMs allowed us to target specific, biomechanically meaningful parameters in the stair negotiation cycle, such as angular velocity, especially within the sagittal plane. In the cycles of stair ascent and descent, both the instances of foot contact with the step and subsequent lift-off were marked by significant retention of hip and knee sagittal angular velocity in PCM I and II. This significance was further highlighted by the range analyses conducted in PCM III, which preserved the ranges of sagittal angular velocities of the hip and knee joints during both ascending and descending tasks. Notably, older adults with disabilities demonstrated lower mean sagittal angular velocities at the hip and knee joints compared to their nondisabled peers. This disparity is visually represented in Figure 5, which indicates that while older adults with disabilities exhibit movement patterns similar to those of nondisabled older adults, they tend to adopt more conservative movement strategies. These findings align with previous aging research, which demonstrated that knee joint angular velocity at the point of maximal knee joint moment demand during the stride cycle was significantly lower in older adults compared to younger adults, a pattern not observed for ankle joint angular velocity [17,48]. These studies also found that variation in knee extension-flexion angles and ankle plantarflexion-dorsiflexion angles during stair ascent [48] and descent [17] was comparable between young and older adults, consistent with our observations among older adults with and without disability. Prior research on stair descent also indicated reduced knee ROM in the sagittal plane and increased hip ROM in the frontal and transverse planes in older adults compared to younger adults [47]. It is hypothesized that older adults may rely more on ankle movements to maintain stability, especially in the frontal plane, as a balance-preserving strategy that can help manage potential mediolateral instability on descending stairs compared with younger adults [12,47]. In the present study, although lower limb joint positions did not appear to be predominant in stair descent, the PCA indicated that older adults with disabilities descended stairs with lower sagittal hip ROM and higher frontal ankle ROM, suggesting that older adults with disabilities further used this strategy compared with those without disability.

Within the F-S and S-F cycles, the hip and knee sagittal angular velocity variables were also retained. Additionally, hip angular velocity in the transverse plane was notably retained at the moments of floor contact and leaving the floor during these transitions. The emergence of variables associated with frontal and transverse planes in F-S and S-F aligns with previous findings indicating that older adults exhibit reduced independent control of adjacent joints during these transitions compared to younger adults [49]. Moreover, the hip angle in the frontal plane was higher in older adults with disability in F-S and S-F. The transition from level gait to stair and the opposite necessitates a shift in motor control between these tasks [15], which seems to be significantly more impaired in older adults with disability.

CoM parameters associated with mediolateral displacement and velocity were retained within the stair cycle. In contrast, during the F-S and S-F cycles, the retained parameters predominantly described vertical displacement and velocity, except for F-S velocity. Buckley et al (2013) [19] proposed that reduced peak downwards CoM velocity of older participants may result from stiffening the limb, prolonging limb flexion when lowering the CoM. This effect may be amplified in older adults with disability, according to our findings. Additionally, Nadeau et al (2003) [46] observed that mediolateral CoM control demands are greater during stair negotiation than during level walking, corroborating the critical role of CoM parameters in influencing stair negotiation dynamics.

Given that stair use in the home environment can help older adults without disability retain the ability to perform IADL [50], these insights carry important implications for the development of targeted interventions aimed at supporting the stability and mobility of older adults, especially those with disability. These findings thus provide clinicians and researchers with actionable information to better address the unique mobility needs of this population, facilitating improvements in both safety and quality of life for older adults navigating stair environments.

### Limitations

Despite the original contributions of this study, certain limitations are noted. Older adults with disabilities take significantly more medication than their counterparts. As the analysis of the medication type was not the object of this study, future studies should address that relationship because stair negotiation is influenced by medication intake [51]. Additionally, due to the structure of our PCA-based analysis and the lack of raw group-wise distributions for each retained PC variable, we were unable to calculate reliable effect sizes post hoc. Nevertheless, this study’s PCA marks initial steps in stair negotiation analysis; future research should explore full waveform data of high-loading parameters to gain more comprehensive insights.

The findings of this study align with established literature on stair negotiation biomechanics in older adults, highlighting compensatory behaviors such as reduced sagittal velocities and conservative movement strategies. The unique contribution of this research lies in the detailed retention of kinematic parameters across various movement planes and phases, offering a more nuanced understanding of stability and movement adaptations in older adults with disability. By focusing on discrete events rather than continuous data, our PCM approach provides a more granular analysis of stair negotiation mechanics, identifying critical sagittal angular velocities of the hip and knee joints during stair ascent and descent, as well as frontal and transverse plane movements of these joints during S-F. Additionally, this approach highlights the potential application of PCMs to the data curves for these parameters, enhancing their utility in both research and clinical contexts.

### Conclusions

Overall, this study examined kinematic differences in joint angles, joint velocities, and CoM displacement and velocity between older adults with and without disabilities during stair ascent and descent. Movements F-S and S-F were also analyzed. PCA of 240 kinematic variables led to the selection of 41 key parameters, mainly related to knee and hip joint angular velocity. In the cycles of stair ascent and descent, both the foot contact with the step and subsequent lift-off were marked by significant retention of hip and knee sagittal angular velocity as well as respective Δvelocity. These parameters were also retained within the F-S and S-F cycles, in addition to the hip angular velocity in the transverse plane at the moments of floor contact and leaving the floor. For the stair cycle, CoM parameters were largely linked to mediolateral displacement and velocity range, while during F-S and S-F, parameters describing vertical displacement and velocity were predominant, except for F-S velocity.

The findings from this study provide valuable insights that could inform disability prevention initiatives. This PCA approach represents a foundational step in understanding stair negotiation in older adults, and further research should focus on full waveform data of high-loading parameters to provide a more comprehensive understanding.

## Supplementary material

10.2196/71530 Multimedia Appendix 1 Table comparing the principal component scores between older adults without and with disability.

## Abbreviations

ADL: activities of daily living
CoM: center of mass
F-S: floor-to-stair transition
IADL: instrumental activities of daily living
KMO: Kaiser-Meyer-Olkin
MMSE: Mini-Mental State Examination
PC: principal component
PCA: principal component analysis
PCM: principal component model
ROM: range of motion
S-F: stair-to-floor transition
SRH: self-reported health status
STROBE: Strengthening the Reporting of Observational Studies in Epidemiology
Δvelocity: angular velocity range
