# Supplementary material for: Principal Component Analysis of Stair Negotiation and Floor Transition Kinematics in Older Adults With and Without Functional Disability: Cross-Sectional Study
Source: JMIR Aging. 2025 Aug 27;8:e71530. doi: 10.2196/71530 (PMC12384677; doi:10.2196/71530)
Supplement: Multimedia Appendix 1 — Table comparing the principal component scores between older adults without and with disability. [file aging-v8-e71530-s001.docx]

**Table S1.** Principal component models (PCM), principal components (PC), variance explained and *P*-value corresponding to a Mann-Whitney U test comparing the PC scores between older adults without (ND) and with (D) disability.

| **Variables included in the PCM** | **PC** | **Variance explained** (%) | **Cumulative variance explained** (%) | **Median and Interquartile Range of Z scores** | | ***P*-value** |
| --- | --- | --- | --- | --- | --- | --- |
|  |  |  |  | **ND** | **D** |  |
|  | | | | | | |
| **Stairs** | | | | | | |
| **PCM I**  Hip, Knee, Ankle Position and Velocity (X, Y, Z) at Contact with step  (36 variables included) | | | | | | |
|  | 1 | 16.40 | 16.40 | -0.06 (1.16) | 0.08 (1.15) | .910 |
|  | 2 | 14.45 | 30.85 | -0.07 (1.37) | 0.21 (1.32) | .311 |
|  | 3 | 9.93 | 40.77 | 0.25 (1.15) | -0.08 (1.38) | .431 |
|  | 4 | 8.11 | 48.88 | -0.15 (1.40) | 0.55 (1.11) | .002 |
|  | 5 | 7.95 | 56.83 | -0.03 (1.21) | 0.25 (2.30) | .747 |
|  | 6 | 6.75 | 63.58 | 0.32 (1.42) | -0.32 (0.89) | .144 |
|  | 7 | 5.34 | 68.92 | -0.36 (1.24) | 0.02 (1.44) | .066 |
|  | 8 | 4.68 | 73.60 | -0.17 (1.21) | 0.07 (1.68) | .534 |
|  | 9 | 3.86 | 77.46 | 0.05 (1.10) | -0.11 (1.57) | .669 |
|  | 10 | 3.57 | 81.04 | -0.19 (1.38) | 0.07 (1.46) | .081 |
|  | 11 | 3.29 | 84.33 | -0.01 (1.46) | -0.06 (1.65) | .910 |
| **PCM II**  Hip. Knee. Ankle Position and Velocity (X, Y, Z) when Leaving the step  (36 variables included) | | | | | | |
|  | 1 | 19.44 | 19.44 | 0.02 (1.11) | -0.17 (1.06) | .364 |
|  | 2 | 15.27 | 34.71 | 0.29(1.16) | -0.50 (1.15) | .008 |
|  | 3 | 11.00 | 45.70 | 0.24 (1.26) | -0.27 (1.24) | .007 |
|  | 4 | 9.20 | 54.90 | -0.01 (1.10) | 0.15 (1.91) | .647 |
|  | 5 | 7.26 | 62.16 | 0.29 (1.11) | -0.15 (1.36) | .216 |
|  | 6 | 6.59 | 68.74 | 0.00 (1.64) | 0.36 (1.05) | .058 |
|  | 7 | 4.94 | 73.68 | 0.12 (1.42) | -0.03 (1.66) | .691 |
|  | 8 | 3.96 | 77.65 | -0.08 (1.57) | 0.33 (1.08) | .341 |
|  | 9 | 3.83 | 81.47 | -0.31 (1.45) | -0.07 (1.21) | .805 |
|  | 10 | 2.95 | 84.42 | -0.06 (1.59) | 0.07 (1.22) | .414 |
| **PCM III**  Hip, Knee, Ankle ROM and ΔVelocity (X, Y, Z)  (36 variables included) | | | | | | |
|  | 1 | 21.18 | 21.18 | 0.29 (1.25) | -0.54 (1.12) | .026 |
|  | 2 | 20.10 | 41.28 | -0.23 (0.66) | -0.04 (0.68) | .270 |
|  | 3 | 11.64 | 52.92 | -0.19 (1.00) | -0.15 (0.77) | .840 |
|  | 4 | 7.03 | 59.95 | 0.05 (1.42) | 0.10 (1.48) | .356 |
|  | 5 | 6.36 | 66.31 | -0.13 (0.96) | -0.34 (1.93) | .691 |
|  | 6 | 5.04 | 71.35 | 0.04 (1.51) | -0.30 (1.20) | .440 |
|  | 7 | 3.60 | 74.95 | -0.26 (1.06) | 0.09 (1.72) | .440 |
|  | 8 | 3.41 | 78.36 | 0.16 (1.61) | 0.09 (1.36) | .505 |
|  | 9 | 3.11 | 81.47 | 0.03 (1.42) | -0.16 (1.34) | .781 |
|  | 10 | 2.81 | 84.29 | -0.28 (1.18) | 0.17 (1.28) | .018 |
| **PCM IV**  CoM Displacement and ΔVelocity  (X, Y, Z) (12 variables included) | | | | | | |
|  | 1 | 32.97 | 32.91 | 0.17 (1.39) | -0.51 (1.37) | .014 |
|  | 2 | 17.28 | 50.25 | 0.10 (1.35) | -0.35 (1.02) | .068 |
|  | 3 | 10.38 | 60.63 | -0.11 (1.36) | 0.13 (1.04) | .495 |
|  | 4 | 10.01 | 70.64 | -0.28 (1.31) | 0.12 (1.43) | .205 |
| **Transitions S-F and F-S** | | | | | | |
| **PCM V**  Hip, Knee, Ankle Position and Velocity (X, Y, Z) at Contact with floor  (36 variables included) | | | | | | |
|  | 1 | 18.07 | 18.07 | -0.03 (1.11) | -0.11 (1.28) | .910 |
|  | 2 | 14.48 | 32.55 | 0.03 (1.29) | -0.23 (1.27) | .574 |
|  | 3 | 10.34 | 42.90 | 0.33 (1.71) | -0.35 (0.99) | .039 |
|  | 4 | 8.44 | 51.34 | 0.05 (1.57) | -0.43 (1.07) | .277 |
|  | 5 | 7.40 | 58.74 | 0.07 (1.60) | -0.10 (1.24) | .505 |
|  | 6 | 6.19 | 64.93 | -0.11 (1.48) | 0.14 (1.47) | .958 |
|  | 7 | 5.00 | 69.93 | -0.12 (1.06) | 0.06 (1.79) | .680 |
|  | 8 | 4.81 | 74.74 | 0.10 (1.18) | 0.06 (1.24) | .725 |
|  | 9 | 3.68 | 78.42 | 0.21 (1.03) | 0.00 (1.49) | .185 |
|  | 10 | 3.02 | 81.44 | -0.07 (1.58) | 0.19 (1.69) | .793 |
| **PCM VI**  Hip, Knee, Ankle Position and Velocity (X, Y, Z) when Leaving the floor  (36 variables included) | | | | | | |
|  | 1 | 19.81 | 19.81 | 0.09 (1.31) | -0.28 (1.14) | .258 |
|  | 2 | 15.93 | 35.73 | -0.17 (1.13) | 0.34(0.90) | .033 |
|  | 3 | 10.06 | 45.79 | -0.41 (1.93) | 0.33 (1.24) | .031 |
|  | 4 | 7.54 | 53.34 | 0.39 (1.65) | -0.09 (1.26) | .333 |
|  | 5 | 6.86 | 60.19 | 0.16 (1.49) | -0.07 (1.57) | .467 |
|  | 6 | 6.42 | 66.61 | -0.07 (1.21) | -0.02 (2.00) | .982 |
|  | 7 | 5.23 | 71.83 | -0.12 (0.91) | 0.09 (1.31) | .887 |
|  | 8 | 4.63 | 76.47 | 0.03 (1.41) | 0.17 (1.05) | .389 |
|  | 9 | 4.18 | 80.65 | -0.19 (1.28) | 0.35 (1.21) | .006 |
|  | 10 | 2.79 | 83.44 | 0.10 (1.35) | 0.01 (1.63) | .680 |
| **PCM VII**  Hip, Knee, Ankle ROM and ΔVelocity (X, Y, Z)  (36 variables included) | | | | | | |
|  | 1 | 25.12 | 25.12 | 0.29 (1.08) | -0.19 (1.39) | .030 |
|  | 2 | 16.62 | 41.74 | 0.09 (1.48) | -0.06 (1.33) | .195 |
|  | 3 | 12.40 | 54.14 | -0.19 (0.52) | -0.42 (1.17) | .647 |
|  | 4 | 7.62 | 61.76 | -0.13 (0.80) | -0.17 (0.79) | .982 |
|  | 5 | 6.54 | 68.30 | -0.14 (0.67) | -0.07 (0.71) | .554 |
|  | 6 | 5.90 | 74.20 | -0.12 (1.24) | -0.15 (0.99) | .725 |
|  | 7 | 4.18 | 78.37 | -0.15 (1.22) | -0.06 (1.44) | .647 |
|  | 8 | 3.64 | 82.02 | -0.31 (1.33) | 0.04 (1.33) | .691 |
| **PCM VIII**  CoM Displacement and ΔVelocity  (X, Y, Z)  (12 variables included) | | | | | | |
|  | 1 | 30.358 | 30.36 | 0.18 (1.13) | -0.31 (1.31) | .083 |
|  | 2 | 19.058 | 49.42 | -0.32 (1.84) | 0.02 (0.98) | .047 |
|  | 3 | 13.274 | 62.69 | -0.26 (1.38) | 0.06 (1.65) | .405 |
